# Vital Signs: Recent Trends in Stroke Death Rates — United States, 2000–2015

**DOI:** 10.15585/mmwr.mm6635e1

**Published:** 2017-09-08

**Authors:** Quanhe Yang, Xin Tong, Linda Schieb, Adam Vaughan, Cathleen Gillespie, Jennifer L. Wiltz, Sallyann Coleman King, Erika Odom, Robert Merritt, Yuling Hong, Mary G. George

**Affiliations:** 1 Division for Heart Disease and Stroke Prevention, National Center for Chronic Disease Prevention and Health Promotion, CDC.

## Abstract

**Introduction:**

The prominent decline in U.S. stroke death rates observed for more than 4 decades has slowed in recent years. CDC examined trends and patterns in recent stroke death rates among U.S. adults aged ≥35 years by age, sex, race/ethnicity, state, and census region.

**Methods:**

Trends in the rates of stroke as the underlying cause of death during 2000–2015 were analyzed using data from the National Vital Statistics System. Joinpoint software was used to identify trends in stroke death rates, and the excess number of stroke deaths resulting from unfavorable changes in trends was estimated.

**Results:**

Among adults aged ≥35 years, age-standardized stroke death rates declined 38%, from 118.4 per 100,000 persons in 2000 to 73.3 per 100,000 persons in 2015. The annual percent change (APC) in stroke death rates changed from 2000 to 2015, from a 3.4% decrease per year during 2000–2003, to a 6.6% decrease per year during 2003–2006, a 3.1% decrease per year during 2006–2013, and a 2.5% (nonsignificant) increase per year during 2013–2015. The last trend segment indicated a reversal from a decrease to a statistically significant increase among Hispanics (APC = 5.8%) and among persons in the South Census Region (APC = 4.2%). Declines in stroke death rates failed to continue in 38 states, and during 2013–2015, an estimated 32,593 excess stroke deaths might not have occurred if the previous rate of decline could have been sustained.

**Conclusions and Implications for Public Health Practice:**

Prior declines in stroke death rates have not continued in recent years, and substantial variations exist in timing and magnitude of change by demographic and geographic characteristics. These findings suggest the importance of strategically identifying opportunities for prevention and intervening in vulnerable populations, especially because effective and underused interventions to prevent stroke incidence and death are known to exist.

## Introduction

Stroke death rates in the United States have declined since at least the 1960s; stroke fell from the third to the fourth leading cause of death in 2008 and to the fifth in 2013. Age-standardized rates among adults aged ≥35 years declined from 315.7 deaths per 100,000 in 1968 to 73.3 per 100,000 in 2015.* The substantial decline in stroke death rates has been attributed to improvements in modifiable stroke risk factors and in stroke treatment and care over time (*1*,*2*). Despite this decline, nearly 800,000 persons in the United States have a new or recurrent stroke each year, and approximately 140,000 stroke victims die; thus, stroke accounts for one in every 20 deaths (*3*). Stroke is also a leading cause of serious long-term disability, with an estimated annual cost of $33.9 billion (*3*).^†^ However, a recent study suggested that the rate of decline in stroke death rates has slowed in recent years, and the rate has even increased slightly since 2013 (*4*). Mortality data from the U.S. National Vital Statistics System from the National Center for Health Statistics were used to examine recent trends in stroke death rates by age, sex, and race/ethnicity at the national level and by census region and state during 2000–2015. The findings of this study will help identify populations that could benefit from interventions to prevent and control modifiable stroke risk factors, further improve the quality of care, and reduce stroke prevalence and mortality.

## Methods

**Data Source.** Stroke death rates were examined among adults aged ≥35 years, who bear the largest burden of stroke (approximately 99% in 2015) and typically share common stroke risk factors. To examine trends in stroke death rates for adults aged ≥35 years, by age, sex, race/ethnicity, U.S. Census region, and state, death data from the U.S. National Vital Statistics System during 2000–2015 with stroke (including all subtypes) reported as the underlying cause of death according to the *International Classification of Diseases, Tenth Revision* (ICD-10; codes I60–I69) were analyzed. Population estimates from the U.S. Census Bureau and CDC’s National Center for Health Statistics for 2000–2015 were used to calculate age-standardized stroke death rates.^§^ Race/ethnicity was categorized into five mutually exclusive groups: non-Hispanic whites (whites), non-Hispanic blacks (blacks), non-Hispanic Asian/Pacific Islanders (A/PI), non-Hispanic American Indian/Alaska Natives, and Hispanics (who could be of any race). State-level analyses were conducted based on the place of residence at death in the United States.

**Statistical analysis.** Age-specific stroke death rates per 100,000 persons by age group (35–54, 55–64, 65–74, 75–84, and ≥85 years) and age-standardized rates by sex, race/ethnicity, census region,^¶^ and state were calculated. Rates were standardized to the 2000 U.S. standard population.

Trend analyses based on the age-standardized or age-specific stroke death rates were conducted to identify different trends in stroke death rates, using Joinpoint software. Joinpoint regression fits a series of joined straight lines on a logarithmic scale to the trend data. These lines, or trend segments, start and end at years where the software detects a statistically significant change in trend. Consequently, trend segments might start and end at different years for each examined variable (e.g., age, race/ethnicity, state, etc.). For each trend segment in the selected model, the annual percent change (APC) was calculated, and the average APC for all years (2000–2015) was obtained as the weighted APC. Because only 16 data points were available for trend analysis, modeling was limited to a maximum of three joinpoints, and the permutation test was used for model selection. Unfavorable changes in the trends were categorized as 1) slowed (a significantly decreasing APC followed by a less negative [significant or nonsignificant] decreasing APC; 2) stalled (a significantly decreasing APC followed by a nonsignificantly increasing APC); or 3) reversed (a significantly decreasing APC followed by a significantly increasing APC (*4*). The number of “excess” stroke deaths associated with the unfavorable changes in trends was estimated in three steps. First, age-, sex-, and race/ethnicity-specific stroke death rates were analyzed using Joinpoint, extrapolating that the stroke death rates would continue to decline through 2015 at the same annual rate as the immediately preceding APC. Second, the age-, sex-, and race/ethnicity-specific populations were multiplied by the extrapolated stroke death rates, to calculate the “expected” number of stroke deaths. Finally, the difference between the observed and extrapolated stroke deaths were calculated by age, sex, and race/ethnicity over time to obtain the number of estimated excess stroke deaths. Because a small number of deaths occurred in the age group 35–54 years, this group was combined with the age group 55–64 years to obtain a stable estimate of excess stroke deaths. Estimated excess stroke deaths during 2013–2015 are reported for better comparability across the groups, noting that the unfavorable changes in trends began in different years for different groups.

## Results

**FIGURE 1:**
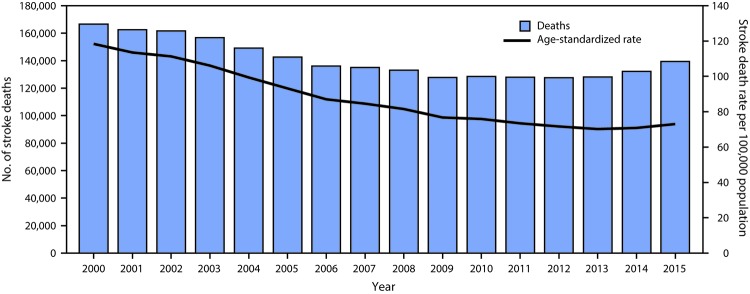
Stroke deaths and age-standardized stroke death rate among adults aged ≥35 years — United States, 2000–2015

Among U.S. adults aged ≥35 years, age-standardized stroke death rates declined 38% from 2000 (118.4 per 100,000 persons) to 2015 (73.3 per 100,000 persons) (Figure 1) with an average APC of -3.1% (Table 1) (Supplementary Table 1, https://stacks.cdc.gov/view/cdc/47567). The mean annual percent decline in stroke death rates changed during 2000–2015: a 3.4% decline per year during 2000–2003, a 6.6% decline per year during 2003–2006, a 3.1% decline per year during 2006–2013, and a nonsignificant 2.5% increase per year during 2013–2015 (Table 1). Although stroke death rates among adults aged 35–54 years declined from 2006 to 2015, for all other age groups and both sexes, and the overall national trend was characterized by stalling declines in the two most recent trend segments. Blacks experienced the highest stroke death rate compared with other racial/ethnic groups, and the stalling of the rate of decline among this group began in 2012. Among Hispanics, the stroke death rate trend reversed in 2013, changing from a 3.6% decline per year during 2000–2013, to a significant 5.8% increase per year during 2013–2015. Stroke death rates continued to decline among American Indian/Alaska Natives during 2000–2015. In the South Census Region, stroke death rate APCs also reversed in 2013, from a 3.3% decline per year during 2006–2013, to a significant 4.2% increase per year during 2013–2015. In the West, Northeast, and Midwest Census Regions, the decline in stroke death rates slowed or stalled in the last trend segment (APC = 0.6% [West], 0.7% [Northeast], and -1.5% [Midwest]). The temporal patterns in national stroke death rates primarily were driven by the rates among adults aged ≥65 years across sex and racial/ethnic groups (Table 1).

**TABLE 1:** Age-standardized stroke death rates and annual percentage change by selected characteristics, adults aged ≥35 years — United States, 2000–2015

Characteristic	No. stroke deaths (age-standardized rate)*		Average APC (95% CI)	Trend segment 1		Trend segment 2		Trend segment 3		Trend segment 4	
	2000	2015	2000–2015	Year	APC (95% CI)	Year	APC (95% CI)	Year	APC (95% CI)	Year	APC (95% CI)
**Total**	**166,611 (118.4)**	**139,367 (73.3)**	**-3.1 (-3.9 to -2.3)**	**2000–2003**	**-3.4 (-5.1 to -1.5)^†^**	**2003–2006**	**-6.6 (-10.2 to -2.8)^†^**	**2006–2013**	**-3.1 (-3.8 to -2.4)^†^**	**2013–2015**	**2.5 (-1.6 to 6.9)**
**Sex**											
Men	64,228 (121.3)	57,750 (73.6)	-3.3 (-4.0 to -2.6)	2000–2003	-3.7 (-5.4 to -2.1)^†^	2003–2006	-6.7 (-10.0 to -3.3)^†^	2006–2012	-3.4 (-4.2 to -2.6)^†^	2012–2015	0.8 (-1.0 to 2.6)
Women	102,383 (114.9)	81,617 (71.8)	-3.1 (-4.0 to -2.2)	2000–2003	-3.2 (-5.1 to -1.2)^†^	2003–2006	-6.5 (-10.4 to -2.4)^†^	2006–2013	-3.2 (-4.0 to -2.5)^†^	2013–2015	2.7 (-1.9 to 7.5)
**Age group (yrs)**											
35–54	8,610 (10.4)	7,095 (8.5)	-1.4 (-1.8 to -1.0)	2000–2006	-0.5 (-1.4 to 0.3)	2006–2015	-2.0 (-2.5 to -1.5)^†^	—^§^	—	—	—
55–64	9,956 (41.0)	12,116 (29.6)	-2.1 (-2.7 to -1.6)	2000–2004	-4.3 (-5.7 to -2.9)^†^	2004–2010	-2.5 (-3.5 to -1.6)^†^	2010–2015	0.1 (-0.8 to 1.1)	—	—
65–74	23,649 (128.6)	20,793 (75.5)	-3.5 (-4.1 to -3.0)	2000–2009	-4.8 (-5.1 to -4.6)^†^	2009–2013	-2.8 (-4.5 to -1.1)^†^	2013–2015	0.9 (-2.4 to 4.3)	—	—
75–84	57,020 (461.3)	38,012 (273.0)	-3.5 (-4.3 to -2.7)	2000–2003	-3.7 (-5.4 to -1.9)^†^	2003–2006	-7.0 (-10.5 to -3.3)^†^	2006–2013	-3.2 (-3.9 to -2.5)^†^	2013–2015	1.3 (-3.0 to 5.9)
≥85	67,376 (1,589.2)	61,351 (975.8)	-3.2 (-4.5 to -2.0)	2000–2003	-2.9 (-5.7 to 0.0)	2003–2006	-8.0 (-13.5 to -2.0)^†^	2006–2013	-3.3 (-4.4 to -2.3)^†^	2013–2015	4.4 (-2.0 to 11.1)
**Race/Ethnicity^¶^**											
White	137,981 (115.2)	106,770 (71.3)	-3.2 (-3.9 to -2.4)	2000–2003	-3.5 (-5.2 to -1.8)^†^	2003–2006	-6.8 (-10.3 to -3.2)^†^	2006–2013	-3.0 (-3.6 to -2.3)^†^	2013–2015	2.5 (-1.7 to 6.7)
Black	18,850 (161.1)	17,593 (102.0)	-3.0 (-3.7 to -2.3)	2000–2002	-2.1 (-6.4 to 2.5)	2002–2012	-4.5 (-5.0 to -4.1)^†^	2012–2015	1.6 (-0.9 to 4.1)	—	—
Hispanic	6,018 (89.7)	9,599 (62.5)	-2.4 (-2.9 to -2.0)	2000–2013	-3.6 (-3.9 to -3.4)^†^	2013–2015	5.8 (2.1 to 9.6)^†^	—	—	—	—
AI/AN	549 (97.2)	634 (62.1)	-3.3 (-3.9 to -2.8)	2000–2015	-3.3 (-3.9 to -2.8)^†^	—	—	—	—	—	—
A/PI	3,213 (103.3)	4,771 (58.5)	-4.1 (-4.7 to -3.6)	2000–2009	-5.3 (-6.0 to -4.6)^†^	2009–2015	-2.3 (-3.5 to -1.2)^†^	—	—	—	—
**Census regions**											
Northeast	29,155 (96.6)	22,195 (60.0)	-3.1 (-4.0 to -2.3)	2000–2003	-3.8 (-5.6 to -2.0)^†^	2003–2006	-6.3 (-10.0 to -2.4)^†^	2006–2013	-2.5 (-3.3 to -1.8)^†^	2013–2015	0.7 (-3.6 to 5.3)
Midwest	40,959 (120.5)	31,240 (73.8)	-3.4 (-3.9 to -3.0)	2000–2009	-4.7 (-5.2 to -4.2)^†^	2009–2015	-1.5 (-2.6 to -0.5)^†^	—	—	—	—
South	62,529 (127.8)	57,142 (82.6)	-2.9 (-3.7 to -2.1)	2000–2003	-3.3 (-5.1 to -1.5)^†^	2003–2006	-6.0 (-9.6 to -2.2)^†^	2006–2013	-3.3 (-3.9 to -2.6)^†^	2013–2015	4.2 (0.1 to 8.5)^†^
West	33,968 (122.7)	28,790 (68.9)	-3.9 (-4.8 to -2.9)	2000–2003	-3.1 (-5.4 to -0.8)^†^	2003–2006	-8.4 (-12.9 to -3.7)^†^	2006–2012	-4.1 (-5.3 to -3.0)^†^	2012–2015	0.6 (-2.0 to 3.3)

**Abbreviations:** A/PI = Asian/Pacific Islander; AI/AN = American Indian/Alaska Native; APC = annual percentage change; CI = confidence interval.

* Per 100,000 persons, standardized to U.S. 2000 population with age groups 35–54, 55–64, 65–74, 75–84 and ≥85 years.

^†^ Significant at p = 0.05.

^§^ Dashes indicate that the best-fit joinpoint model did not include that trend segment.

^¶^ Whites, blacks, American Indians/Alaska Natives, and Asian/Pacific Islanders are non-Hispanic. Hispanic persons might be of any race.

An estimated 32,593 excess stroke deaths might have occurred because of unfavorable changes in the rate of decline in stroke mortality during 2013–2015. Among the estimated excess stroke deaths, nearly one third (10,269; 32%) occurred among adults aged 35–64 years (Table 2) (Supplementary Table 2, https://stacks.cdc.gov/view/cdc/47568).

**TABLE 2:** Observed, expected, and estimated number of excess stroke deaths by age, sex, and race/ethnicity — United States, 2013–2015

Characteristic	Stroke deaths		
	Observed	Expected*	Excess^†^ (% of total)
**Total**	**378,787**	**364,194**	**32,593 (100)**
**Age group (yrs)**			
35–64	44,843	34,575	10,269 (32)
65–74	54,693	51,314	3,379 (10)
75–84	106,316	100,081	6,235 (19)
≥85	172,935	160,226	12,709 (39)
**Sex**			
Men	160,795	141,267	19,528 (60)
Women	217,992	204,927	13,065 (40)
**Race/Ethnicity^§^**			
White	308,396	285,170	23,226 (71)
Black	43,870	38,030	5,840 (18)
Hispanic	21,823	2,858	2,858 (9)
AI/AN	—^¶^	—	—
A/PI	4,698	4,029	669 (2)

**Abbreviations:** A/PI = Asian/Pacific Islander; AI/AN = American Indian/Alaska Native.

* The expected number of stroke deaths were obtained by 1) assuming that the age-, sex-, and racial/ethnic-specific stroke mortality rates would continue to decline through 2015 at the annual rate of the immediately preceding APC as identified by the Joinpoint analysis, and 2) multiplying the age-, sex-, and racial/ethnic-specific population with the assumed age-, sex-, and racial/ethnic-specific stroke death rates for each year.

^†^ Excess stroke deaths were calculated by 1) estimating the age-, sex-, and race/ethnicity-specific stroke death rates using Joinpoint, assuming the stroke death rates would continue to decline through 2015 at the annual rate of the immediately preceding APC, 2) calculating the “expected” number of stroke death by multiplying the age-, sex-, and race/ethnicity-specific population by the assumed stroke death rates, and 3) calculating the excess stroke deaths based on the difference between the observed and expected stroke deaths by age-, sex-, and race/ethnicity over time. The excess stroke deaths during 2013–2015 were reported for better comparability across the groups because the starting year of the unfavorable changes in trend might be different for different groups.

^§^ Whites, blacks, American Indians/Alaska Natives and Asian/Pacific Islanders are non-Hispanic; Hispanic persons might be of any race.

^¶^ Excess deaths were not calculated for this group because the trend did not change during the study period.

**FIGURE 2:**
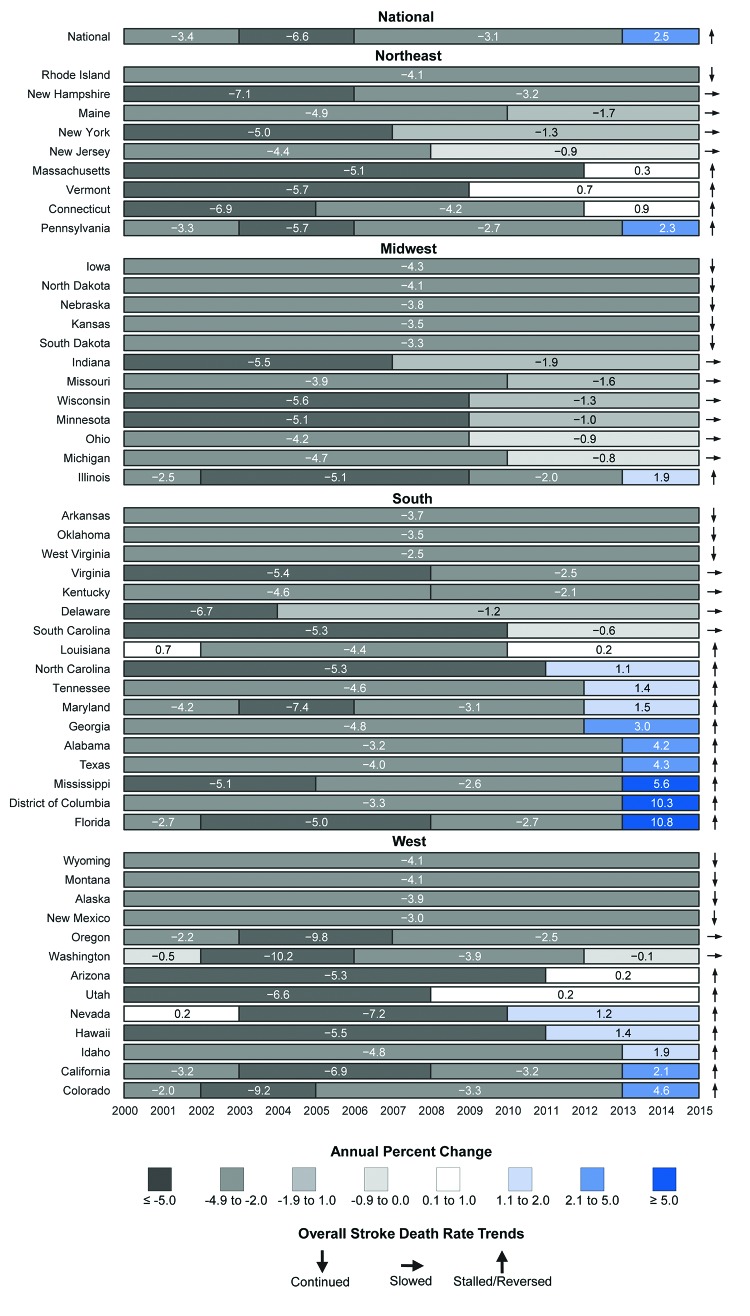
Trends in age-standardized stroke death rates among adults aged ≥35 years, by state and census region — United States, 2000–2015 * Continued: significant decrease in stroke death rate continued over the period. ^†^ Slowed: significant decrease in stroke death rate followed by a less negative decreasing (significant or nonsignificant) trend segment. ^§^ Stalled/reversed: significant decrease in stroke death rate followed by a nonsignificant (stalled) or significant (reversed) increasing trend segment (final Joinpoint trend segment Annual Percent Change >0).

Nationally, 38 (75%) states, including eight of nine states in the Northeast Census Region, seven of 12 in the Midwest, 14 of 17 in the South, and nine of 13 in the West, experienced a slowing, stalling, or reversing in the decline in stroke death rates during 2000–2015 (Supplementary Figure, https://stacks.cdc.gov/view/cdc/47566). In Florida, the decline in the stroke death rate reversed during 2013–2015, with a significant increase (10.8% per year) in the stroke death rate (Figure 2) (Supplementary Table 3, https://stacks.cdc.gov/view/cdc/47569).

## Conclusions and Implications for Public Health Practice

The unfavorable changes in declines in stroke death rates identified in this analysis at the national level and among various demographic groups and geographic areas are consistent with other recent U.S. studies (*4*,*5*). Reasons for the slowing, stalling, and reversing in declines in stroke death rates are not clear. These changes could be related to adverse changes in the prevalence or management of stroke risk factors that might increase stroke incidence and other time-limited factors, such as complications of a severe influenza season, as occurred with drifted H3N2 influenza in 2014–15 (*6*,*7*). It is possible that changes in some stroke risk factors, including increased prevalence of obesity (*8*,*9*), diabetes (*10*), unhealthy diets, and physical inactivity (*11*,*12*), over the past few decades are contributing to the slowing of the decline. Obesity is recognized as a major cause of hypertension, which is the single most important modifiable risk factor for stroke (*13*). Despite recent improvements, nearly half of the 75 million U.S. adults with hypertension do not have their blood pressure under control (*14*,*15*). Recent studies have reported that younger adults have experienced a significant increase in both stroke hospitalizations and in associated stroke risk factors (e.g., hypertension, obesity, diabetes, lipid disorder, and tobacco use) documented at the time of the acute stroke hospitalization during the past 15 years (*16*–*18*). These changes in modifiable stroke risk factors might present new challenges for stroke prevention and for maintaining a sustained decline in stroke mortality in the United States (*19*–*21*).

The observed unfavorable changes in stroke mortality declines could be related to differences in stroke treatment and care among certain population subgroups, leading to disparate increases in stroke case fatality rates (*1*); however, recent studies suggest that the racial and regional disparities in stroke mortality are driven by differences in stroke incidence (*22*,*23*). Since 1950, other periods of stagnation in age-standardized stroke death rates followed by subsequent decline have occurred (*24*). Thus, the recent changes could reflect patterns similar to those that have previously occurred, which were then followed by substantial declines.

Approximately 7 million Americans aged ≥20 years report having had a stroke, yet approximately 80% of strokes are preventable (*1*). Numerous randomized controlled trials, observational studies, and interventions have demonstrated the effectiveness of lifestyle changes, improving the quality of acute stroke care, and secondary prevention in reducing stroke incidence or mortality, disability, and health care costs.**

Recognizing the signs and symptoms of a stroke and knowing the importance of calling 9-1-1 is critical to ensuring that stroke patients receive the best care as quickly as possible. Stroke symptoms tend to occur suddenly and include sudden onset of weakness or numbness on one side of the body, sudden confusion, trouble speaking or understanding, sudden trouble seeing in one or both eyes, sudden trouble walking, dizziness or loss of balance, or a sudden severe headache. CDC’s Paul Coverdell National Acute Stroke Program (https://www.cdc.gov/dhdsp/programs/stroke_registry.htm) is working closely with partners in health care to enhance the quality of stroke care through data-driven quality improvement in approximately 570 hospitals, which have treated >620,000 acute stroke patients in the United States since 2005. With better recognition of stroke by the general public and emergency medical services, better care provided by emergency medical services and in the hospital, and the initiation of secondary stroke prevention, the Coverdell program is developing coordinated systems of care to reduce stroke-related death and disability. In addition, the goal of the Million Hearts initiative,^††^ co-led by CDC and the Centers for Medicare & Medicaid Services and supported by many public and private partners, is to prevent 1 million heart attacks and strokes by 2022. This goal can be achieved by reducing sodium intake, tobacco use, and physical inactivity, and improving implementation of the ABCS (aspirin when appropriate, blood pressure control, cholesterol management, and smoking cessation), cardiac rehabilitation, and patient engagement, with a heightened focus on priority populations at high risk for heart disease and stroke.

The findings in this report are subject to at least six limitations. First, the underlying cause of death on death certificates might be subject to misclassification, as well as changes or improvements in cause of death identification (*1*). Second, age-standardized stroke death rates do not represent actual stroke death rates; they were appropriate for comparisons across populations and over time, but will vary more from the unadjusted rate as the population distribution changes over time. To address this concern, age-specific rates were also provided. Third, Joinpoint with permutation tests were used for the trend analyses; selecting different models that use different statistical tests might result in different trend patterns. Fourth, the lower number of stroke deaths in some states might affect the detection of trends. Fifth, excess stroke deaths were estimated by assuming stroke death rates would continue to decline through 2015 at the annual rate of the most recent APC, and represents the hypothetical achievable reduction in stroke deaths. Finally, in light of evidence supporting a role for influenza infection in the development of cardiovascular events (*25*,*26*), it is unclear what effect the severe influenza seasons in 2012–13 and 2014–15 (https://gis.cdc.gov/grasp/fluview/mortality.html) might have had on stroke mortality in recent years.

The substantial decline in stroke death rates during the past four decades has slowed, stalled or, in some cases, reversed in recent years, and substantial variations exist in the timing and magnitude of this unfavorable change among subpopulations and states. These findings emphasize the importance of strategically identifying disparities in specific risk factors, incidence, and geography that might be driving the unfavorable changes in decline, so that targeted interventions can be implemented to prevent strokes in vulnerable populations.

**Key Points**

• After more than 4 decades of decline, stroke death rates in the United States have declined more slowly, stalled, or reversed among some subpopulations in recent years.

• Trends in stroke death rates reversed in 2013 among Hispanics and in the South Census Region, where significant declines from year to year changed to significant increases during 2013–2015.

• Thirty-eight states had an unfavorable change in the rate of decline in stroke death rates during 2000–2015.

• An estimated 30,000 excess stroke deaths might have occurred because of the unfavorable changes in the rate of decline in stroke mortality during 2013–2015.

• The findings emphasize the importance of continuing surveillance of stroke and strategically identifying disparities in specific risk factors, incidence, and geography that might be driving the unfavorable changes in the rate of decline so that targeted interventions can be implemented to prevent strokes in vulnerable populations.

• Additional information is available at https://www.cdc.gov/vitalsigns/.

## Footnotes

* American Heart Association annual Heart Disease and Stroke Statistics updates. https://www.heart.org/HEARTORG/General/Heart-and-Stroke-Association-Statistics_UCM_319064_SubHomePage.jsp; CDC WONDER Underlying Cause-Of-Death. https://wonder.cdc.gov/mortSQL.html.

† Direct and indirect costs.

§ Bridged-race postcensal estimates were used for population estimates. https://www.cdc.gov/nchs/data/series/sr_02/sr02_135.pdf.

¶ https://www.census.gov/geo/reference/gtc/gtc_census_divreg.html.

** https://www.cdc.gov/stroke/healthy_living.htm.

†† https://millionhearts.hhs.gov.
